# Outbreak of West Nile virus infection, Volgograd Region, Russia, 1999.

**DOI:** 10.3201/eid0701.010118

**Published:** 2001

**Authors:** A E Platonov, G A Shipulin, O Y Shipulina, E N Tyutyunnik, T I Frolochkina, R S Lanciotti, S Yazyshina, O V Platonova, I L Obukhov, A N Zhukov, Y Y Vengerov, V I Pokrovskii

**Affiliations:** Central Institute of Epidemiology, Novogireevskaya Str. 3A, Moscow 111123, Russia. plt@online.ru

## Abstract

From July 25 to October 1, 1999, 826 patients were admitted to Volgograd Region, Russia, hospitals with acute aseptic meningoencephalitis, meningitis, or fever consistent with arboviral infection. Of 84 cases of meningoencephalitis, 40 were fatal. Fourteen brain specimens were positive in reverse transcriptase-polymerase chain reaction assays, confirming the presence of West Nile/Kunjin virus.


### Outbreak of West Nile Virus Infection, Volgograd Region, Russia, 1999

**Alexander E. Platonov,* German A. Shipulin,* Olga Yu. Shipulina,* Elena N. Tyutyunnik,† Tatyana I. Frolochkina,‡ Robert S. Lanciotti,§ Svetlana Yazyshina,¶ Olga V. Platonova, * Igor L. Obukhov,¶ Alexander N. Zhukov,** Yury Ya. Vengerov,† and Valenin I. Pokrovskii***

*Central Institute of Epidemiology, Moscow, Russia; †Moscow State University for Medicine and Dentistry, Russia; ‡Ministry of Public Health, Moscow, Russia; §Centers for Disease Control and Prevention, Fort Collins, Colorado, USA; ¶Russian State Institute for Control of Veterinary Products, Moscow, Russia; **Center of Sanitary and Epidemic Control for Volgograd Region, Volgograd, Russia

From July 25 to October 1, 1999, 826 patients were admitted to Volgograd Region, Russia, hospitals with acute aseptic meningoencephalitis, meningitis, or fever consistent with arboviral infection. Of 84 cases of meningoencephalitis, 40 were fatal. Fourteen brain specimens were positive in reverse transcriptase-polymerase chain reaction assays, confirming the presence of West Nile/Kunjin virus.

West Nile (WN) virus is a member of the Japanese encephalitis (JE) antigenic complex of the genus Flavivirus, family Flaviviridae. Mosquito-borne WN virus fever is endemic in Africa, the Middle East, and Southwest Asia. The antigenically and genetically related Kunjin virus is a WN virus counterpart in Australia and Southeast Asia and has recently been taxonomically classified as a subtype of WN virus. Until recently, WN virus infection in humans was considered a relatively mild, influenzalike disease with full recovery, although occasionally (<15% of cases) acute aseptic meningitis or encephalitis occurred (1). No large outbreak of WN virus fever was reported in Europe until August and September 1996, when more than 500 clinical cases were observed in Romania (Bucharest region), with high rates of neurologic disorders and death (up to 10%) (2). WN virus had never been detected in the Western Hemisphere until August 1999, when an outbreak of human WN encephalitis in New York City (56 confirmed cases, 7 deaths) coincided with unusual deaths in crows and exotic birds (3-5).

Address for correspondence: A.E. Platonov, Central Institute of Epidemiology, Novogireevskaya Str. 3A, Moscow 111123, Russia; Fax: 7-095-305-5423; e-mail: plt@online.ru.

### The Study

In August and September 1999, an outbreak of acute viral infection consistent with arboviral infection occurred in the Volgograd Region, Russia. Epidemiologic and clinical data were collected and analyzed in the Center of Sanitary and Epidemic Control for the Volgograd Region in collaboration with the Commission of the Russian Ministry of Public Health. From July 25 to October 1, 826 patients were admitted to area hospitals with the clinical diagnosis of acute aseptic meningoencephalitis (code A86, ICD-10; 84 patients), acute aseptic meningitis (code A87.9; 308 patients), or acute viral infection with fever (code B34.9; 347 patients). Serum samples from 318 patients were tested for WN virus antibody by immunoglobulin (Ig)M-capture enzyme-linked immunosorbent assay (ELISA) and indirect IgG ELISA (2,6); 183 (58%) samples demonstrated a level of anti-WN virus IgM indicative of acute infection. This proportion was approximately the same in patients with aseptic meningoencephalitis, aseptic meningitis, and acute fever. These 183 cases were considered serologically confirmed WN virus cases; all 826 cases were considered clinically compatible WN virus cases. The total number of suspected overt human WN virus cases was estimated to be 480.

Volgograd City (population 1 million) is located on the west bank of the Great Volga River (latitude 48°N, longitude 44°E) in the Russian steppe; Volzskii City (population 300,000) is on the opposite bank. Approximately 65% and 30% of WN virus cases were from Volgograd and Volzskii, respectively; the rest occurred in the rural region around Volgograd, near the Volga River or its tributaries. The male:female infection ratio was 1:1. The incidence of infection was age specific; more than 50% of patients were ≥50 years of age and less than 15% were ≤15 years. The epidemic peaked between August 21 and August 25, waning with the onset of cooler temperatures in late September. Figures were similar for serologically confirmed and clinically probable WN virus cases.

The clinical characteristics of the Volgograd WN virus epidemic differed somewhat from those of previous outbreaks (1). In Volgograd, the disease was generally more severe, with a higher than normal case-fatality rate. The central nervous system was usually involved, and acute aseptic meningitis or encephalitis was frequently observed. Rash and conjunctivitis were rarely observed. Abdominal pain, diarrhea, respiratory symptoms, and lymphadenopathy were rare or absent. As in other WN virus epidemics, clinical features included abrupt onset of disease, asthenia, high fever (up to 39°C-40°C), headache, and vomiting.

Of the 84 cases of acute aseptic meningoencephalitis, 40 were fatal (7). Autopsies of laboratory-confirmed WN meningoencephalitis cases revealed perivascular hemorrhages, ectasis of ventriculi of the brain, foci of encephalomalacia, dislocation of the brain trunk (30% of cases), and hydropericarditis with flabbiness of the cardiac muscle. Microscopy findings included signs of focal encephalitis and vasculitis, lymphocytic perivascular inflammatory infiltration, profound degenerative and necrobiotic changes of ganglion cells in the cerebral cortex, and signs of brain edema, as well as parenchymal myocarditis (stromal edema, degeneration of myocytes, foci of myolysis, and fragmentation of myofibrils). Thirty (75%) of the patients who died were >60 years of age.

We have developed reverse transcriptase-polymerase chain reaction (RT-PCR) assays specific for WN/Kunjin genome and a consensus assay for the detection of all flavivirus genomes (5,8, 9). Two pairs of oligonucleotide primers (WN11/WN2, WN1/WN2) were designed to hybridize to a relatively conserved region within the envelope (E) gene of WN/Kunjin viruses. The expected amplification product was 222 base pairs long (WN1, 5'- AGG, GGC, CAC, CCA, GGC, TGG, AAG, ATT, CA- 3'; WN11, 5'-TGG, GGC, CAC, TCA, GGC, AGG, GAG, ATT, CA-3'; WN2, 5'-CAC, GTG, GTG, CTT, CCA, GCA, CTG, CTC, CA-3'). Another pair of primers, FLV1/FLV2, was designed to hybridize to conserved regions within the RNA replicase (NS5) gene of a wide variety of flaviviruses, amplifying nearly a 220-bp fragment (FLV1, 5'-GGI, AGC, AGI, GCC, ATI, TGG, T(A/T)C, ATG, TGG - 3'; FLV-2, 5'-C(G/T)I, GTG, TCC, CAI, CCI, GCI, GTG, TCA, TC-3').

Brain tissue samples taken at autopsy from 14 patients with meningoencephalitis were subjected to RT-PCR with primer pairs WN11/WN2, WN1/WN2, and FLV1/FLV2. The samples and corresponding viral RNA/cDNA preparations are designated below as Volgograd-1999-1, Volgograd-1999-2, and the like. Two WN virus strains isolated in 1967-1970 in the Republic of Azerbaijan (former Soviet Union) and the prototype JE strain isolated in Tokyo were used as control templates in the RT-PCR assay. All 14 brain samples were strongly positive in the RT-PCR assays with WN11/WN2, WN1/WN2, and FLV1/FLV2, confirming the presence of WN/Kunjin sequences. The WN-Azerbaijan-1967 and WN-Azerbaijan-1970 control RNA preparations were positive only with WN11/WN2 and FLV1/FLV2 primer pairs, indicating that some differences in the corresponding region of the E gene from the old and new “Russian” WN virus strains were likely. As expected, the JE-Tokyo-1935 RNA preparation was negative in the WN-specific RT-PCR assay, but positive with FLV1/FLV2 primers.

The amplification products obtained from the E and NS5 genes from seven patients (#1, 3, 7, 11, 12, 13, 14) and from the reference WN-Azerbaijan-1967 and WN-Azerbaijan-1970 strains were subjected to DNA sequencing. The sequences obtained from the Volgograd patients were identical, suggesting infection with a single WN virus strain.

The E sequences of the Volgograd and Azerbaijan WN viruses were aligned with each other and with 38 other WN/Kunjin strains, by using CLUSTAL W alignment software. The NS5 sequences of the Volgograd and Azerbaijan WN viruses were aligned with 16 other flavivirus strains by CLUSTAL W. The high level of sequence similarity confirmed the WN virus source of the Volgograd cases (Table). Phylogenetic trees of WN/Kunjin viruses and all flaviviruses derived from E and NS5 gene sequences (5,8-10) have been previously described. We used smaller gene fragments, 165 bases of E gene and 147 bases of NS5 gene; however, the deduced phylogenetic trees were practically the same. Therefore, we limited the analysis to the most similar strains, including the epidemiologically important WN-New-York-1999 and WN-Romania-1996 strains, and some representatives of other taxonomic subdivisions. For comparison, the designation of strains coincides with the designation in publications where the additional details of strain history are given (5,10).

The Volgograd and old Azerbaijan WN virus strains clearly belonged to "lineage 1" of WN virus isolates (5,9-10). Within lineage 1, the Volgograd patient strains were most closely related to the current Kenya and Senegal strains and the Romanian mosquito isolate (the identical E gene fragment of 165 nucleotides [nt]). The Azerbaijan isolates were more closely related to

Table. Percentage identity of nucleotide sequence for E and NS5 gene fragments among West Nile/Kunjin viruses

| Virus strain | WN-Romania-1996M | WN-Kenya-1998 and WN-Senegal-1993 | WN-New York-1999F and WN-New York-1999H | WN-Azerbaijan-1967 | WN-Azerbaijan-1970 | WN-Romania-1996H | WN-Egypt-1951 | Kunjin-Aust-1960 | WN-Nigeria (Wengler) |
|---|---|---|---|---|---|---|---|---|---|
| WN-Volgograd-1999-1 and WN-Volgograd-1999-11* | 100 | 100<br>96.6 | 98.2<br>94.6 | 93.9<br>95.2 | 93.9 | 93.9<br>92.5 | 92.3<br>85.0 | 86.7<br>78.2 | 74.5 |
| Romania-1996M | | 100 | 98.2<br>96.6 | 93.9<br>94.6 | 93.9<br>95.2 | 93.9 | 92.3<br>92.5 | 86.7<br>85.0 | 74.5<br>78.2 |
| WN-Kenya-1998 and WN-Senegal-1993 | | | 98.2 | 93.9 | 93.9 | 93.9 | 92.3 | 86.7 | 74.5 |
| WN-New York-1999F and WN-New York-1999H | | | | 95.8<br>95.2 | 95.8<br>94.6 | 95.8 | 94.5<br>93.2 | 87.3<br>85.7 | 75.8<br>78.9 |
| WN-Azerbaijan-1967 | | | | | 100<br>99.3 | 100 | 97.6<br>98.0 | 88.5<br>87.8 | 75.8<br>78.2 |
| WN-Azerbaijan-1970 | | | | | | 100 | 97.6<br>97.3 | 88.5<br>88.4 | 75.8<br>78.2 |
| WN-Romania-1996H | | | | | | | 97.6 | 88.5 | 75.8 |
| WN-Egypt-1951 | | | | | | | | 87.9<br>87.1 | 75.8<br>76.2 |
| Kunjin-Aust-1960 | | | | | | | | | 75.1<br>72.8 |
| WN-Nigeria (Wengler) | | | | | | | | | |

*First lines in a row = percentage identity of nucleotide sequence for E gene fragment; second lines in a row = percentage identity of nucleotide sequence for NS5 gene fragment (if available). The strains with identical sequences of E and NS5 fragments are placed in the same row or column.

WN-Egypt-1951 strain and the Romanian human isolate (Figure). The New York-1999 isolate differed from the Volgograd isolates in 3 nt positions and from the Azerbaijan isolates in 7 nt positions. All 10 polymorphic sites in sequences of Volgograd, New York, Azerbaijan, and Romania strains were in the third codon position, producing silent mutations. WN-Volgograd-1 differed from WN-Egypt-1951 in 1 amino acid (aa) (Leu vs. Trp), from Kunjin in 1 aa (Asn vs. Ser) and from WN-Nigeria in 5 aa within the E fragment of 55 aa.

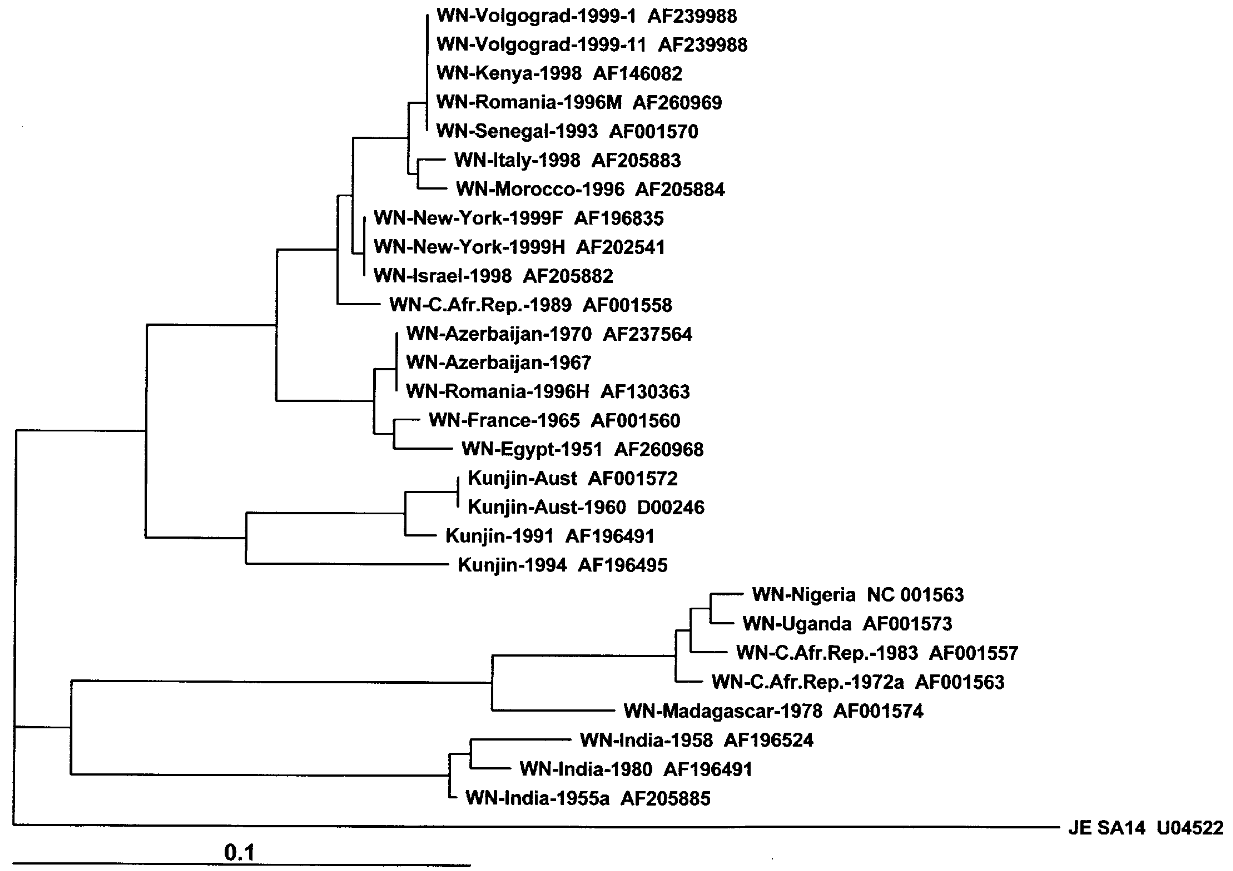

Figure. Phylogenetic trees based on nucleic sequence data of E-glycoprotein gene fragment of 165 bp. The trees were constructed with the program CLUSTAL by using the neighbor joining method of Saitou and Nei with bootstrapping. Tree is rooted by using Japanese encephalitis sequence as an outgroup. The designation of isolates corresponds to that in publications (5,10), where details of isolate history are given. Alignments used for analysis are available upon request from the authors.

[a]WN virus strains used in phylogenetic analysis. WN-Volgograd-1999-1 = human brain tissue from female patient, Volgograd, Russia, GenBank accession # AF239988 (E gene fragment) and #AF239990 (NS5 gene fragment); WN-Kenya-1998 = strain KN3829 isolated from *Culex univittatus*, # AF146082; WN-Senegal-1993 = strain SEN-ArD93548 isolated from *Culex neavei*, # AF001570; WN-Romania-1996M = strain RO97-50 isolated from *Culex pipiens* pool, # AF260969; WN-New-York-1999F = strain NY99-flamingo382-99 isolated from Bronx Zoo flamingo 1999, # AF196835; WN-New-York-1999H = strain HNY1999 isolated from total human brain RNA, # AF202541; WN-Azerbaijan-1967 = isolated from a bird, # AF241822 (NS5 gene fragment); WN-Azerbaijan-1970 = strain A-72 isolated from a tick *Ornitodorus coniceps,* # AF241821 (NS5 gene fragment) and # AF237564 (E gene fragment); WN-Romania-1996H = strain RO96-1030 isolated from human CSF, # AF130363; WN-Egypt-1951 = strain Eg101, #AF260968; Kunjin-Aust-1960 = Kunjin virus MRM61C, # D00246; WN-Nigeria = West Nile virus, # M12294 and #NC_001563.

Although a few sequences of WN virus NS5 gene were available, the data confirmed that the WN virus strains of lineage 1 were more close to each other (the differences in 0-11 nt of 147) than to Kunjin, and especially distant from WN-Nigeria (Wengler) strain (Table). Again, all 10 polymorphic sites in sequences of lineage 1 strains corresponded to the silent mutations only. WN-Volgograd-1 differed from Kunjin in 1 aa (Lys vs. Arg) and from WN-Nigeria in 3 aa (Arg vs. Lys, Arg vs. Lys, and Ile vs. Val) but differed, for example, from Saint Louis (AF013416) in 11 aa within the NS5 fragment of 48 aa. The isolation of WN virus from one of our clinical brain samples, Volgograd-1999-4, will make complete genome sequencing and further virologic investigations possible (11).

### Conclusions

Our data, together with those of previous publications, document several outbreaks of emerging WN virus infection in regions where this disease was not found or was rarely found. (There were a few isolations of WN virus in Romania and the former Soviet Union before 1996-99.) Some isolates demonstrate a high degree of similarity (New York-1999 and Israel-1998; Volgograd-1999, Romania-1996-mosquito isolate, Kenya-1998, and Senegal-1993; Azerbaijan-1967 and Romania-1996-human isolate). Moreover, the last three large outbreaks were caused by genetically similar strains (WN-Romania-1996, WN-New York-1999, and WN-Volgograd-1999), indicating the wide circulation and emergence of potentially epidemic strains of WN virus. All three cities, Bucharest, New York, and Volgograd, are located near large bodies of water and on bird migration pathways and all had unusually dry summers the year of the outbreak (12). Some clinical characteristics of the recent WN virus epidemics were unexpected, such as the high rate of neurologic disorders and death. These unusual characteristics may be due to the expansion of new pathogenic WN virus strain(s) or to the peculiarities of the human host response.

### Acknowledgments

We thank DK Lvov, VL Gromashevskii, AM Butenko, S. Ya. Gaidamovitch, O Vyshemirskii, and VF Larichev for providing the reference WN virus strains and helpful discussions; DJ Gubler, J LeDuc, and P Henry for arranging research collaboration between the Central Institute of Epidemiology, Moscow, and the Centers for Disease Control and Prevention; and NV Rusakova, EM Krasnova, VA Petrov, and AM Alyushin for providing human specimens for testing, and epidemiologic and clinical information for analysis.

Dr. Platonov is head of a laboratory at the Central Institute of Epidemiology, Russia. His scientific interests include the epidemiology, diagnosis, and pathogenesis of bacterial and viral meningitis.

### References

1. Hubálek Z, Halouzka J. West Nile fever—a reemerging mosquito-borne viral disease in Europe. Emerg Infect Dis 1999;5:643-50.
2. Tsai TF, Popovici F, Cernescu C, Campbell GL, Nedelcu NI. West Nile encephalitis epidemic in southeastern Romania. Lancet 1998;352:767-71.
3. CDC. Update: West Nile virus encephalitis—New York, 1999. Morb Mortal Wkly Rep 1999;48:944-6.
4. Anderson JF, Andreadis TG, Vossbrinck CR, Tirrell S, Wakem EM, French RA, et al. Isolation of West Nile virus from mosquitoes, crows, and a Cooper's hawk in Connecticut. Science 1999;286:2331-3.
5. Lanciotti RS, Roehrig JT, Deubel V, Smith J, Parker M, Steele K, et al. Origin of the West Nile virus responsible for an outbreak of encephalitis in the northeastern U.S. Science 1999;286:2333-7.
6. Lvov DR, Butenko AM, Gaidamovitch SY, Larichev VPh, Leschinskaya EV, Zhukov AN, et al. Epidemic outbreak of meningitis and meningoencephalitis in Krasnodar territory and Volgograd region provoked by of West Nile fever virus. Vopr Virusol 2000;45:37-8.(In Russian).
7. Vengerov YuYa, Frolochkina TI, Zhukov AN, Shipulin GA, Shipulina OYu, et al. West Nile virus infection as clinical and epidemiological problem. Epidemiology and Infectious Diseases 2000;4:27-31.(In Russian).
8. Kuno G, Chang GJ, Tsuchiya KR, Karabatsos N, Cropp CB. Phylogeny of the genus Flavivirus. J Virol 1998;72:73-83.
9. Berthet FX, Zeller HG, Drouet MT, Rauzier J, Digoutte JP, Deubel V. Extensive nucleotide changes and deletions within the envelope glycoprotein gene of Euro-African West Nile viruses. J Gen Virol 1997;8(Pt 9):2293-7.
10. Savage HM, Ceianu C, Nicolescu G, Karabatsos N, Lanciotti R, Vladimirescu A, et al. Entomologic and avian investigations of an epidemic of West Nile fever in Romania in 1996, with serologic and molecular characterization of a virus isolate from mosquitoes. Am J Trop Med Hyg 1999;61:600-11.
11. Lvov DR, Butenko AM, Gromashevsky VI, Larichev VPh, Gaidamovitch SYa, Vyshemirsky OI, et al. Isolation of two strains of West Nile virus during an outbreak in Southern Russia, 1999. Emerg Infect Dis 2000;6:373-6.
12. Han LL, Popovici F, Alexander Jr JP, Laurentia V, Tengelsen LA, Cernescu C, et al. Risk factors for West Nile virus infection and meningoencephalitis, Romania, 1996. J Infect Dis 1999;179:230-3.
